# Discovery of Dual‐Functional Amorphous Titanium Suboxide to Promote Polysulfide Adsorption and Regulate Sulfide Growth in Li–S Batteries

**DOI:** 10.1002/advs.202200958

**Published:** 2022-06-05

**Authors:** Donghee Gueon, Jisu Yoon, Jinhan Cho, Jun Hyuk Moon

**Affiliations:** ^1^ Department of Chemical and Biomolecular Engineering Institute of Emergent Materials Sogang University Baekbeom‐ro 35, Mapo‐gu Seoul 04107 Republic of Korea; ^2^ Department of Chemical and Biological Engineering Korea University 145 Anam‐ro, Seongbuk‐gu Seoul 02841 Republic of Korea; ^3^ KU‐KIST Graduate School of Converging Science and Technology Korea University 145 Anam‐ro, Seongbuk‐gu Seoul 02841 Republic of Korea

**Keywords:** amorphous titanium suboxides, DFT calculations, high sulfur loading, lithium sulfide clustering, lithium–sulfur batteries

## Abstract

Lithium‐sulfur (Li–S) batteries are promising as next‐generation energy storage systems. Adsorbents for sulfide species are favorably applied to the cathode, but this substrate often results in a surface‐passivating lithium sulfide(Li_2_S) film with a strong adsorption of Li_2_S. Here, an amorphous titanium suboxide (a‐TiOx) is presented that strongly adsorbs lithium polysulfides (Li_2_S_x_, x < 6) but relatively weakly adsorbs to Li_2_S. With these characteristics, the a‐TiO_x_ achieves high conversion of Li_2_S_x_ and high sulfur utilization accompanying the growth of particulate Li_2_S. The DFT calculations present a mechanism for particulate growth driven by the promoted diffusion and favorable clustering of Li_2_S. The a‐TiO_x_‐coated carbon nanotube‐assembled film (CNTF) cathode substrate cell achieves a high discharge capacity equivalent to 90% sulfur utilization at 0.2 C. The cell also delivers a high capacity of 850 mAh g^–1^ even at the ultra‐high‐speed of 10 C and also exhibits high stability of capacity loss of 0.0226% per cycle up to 500 cycles. The a‐TiO_x_/CNTF is stacked to achieve a high loading of 7.5 mg S cm^–2^, achieving a practical areal capacity of 10.1 mAh cm^–2^.

## Graphical abstract

Amorphous titanium suboxide (a‐TiO_x_), which strongly adsorbs lithium polysulfides (Li_2_S_x_, x<6) but relatively weakly to lithium sulfide (Li_2_S), is introduced for the cathode of lithium‐sulfur (Li–S) battery. The a‐TiO_x_ substrate cathode achieves a high sulfur utilization rate of 90% at 0.2 C by high conversion of Li_2_S_x_ and growth of particulate Li_2_S.

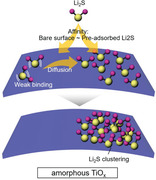

## 1 Introduction

Lithium‐sulfur (Li–S) batteries are attracting attention as a next‐generation energy storage system to replace lithium‐ion batteries due to their high theoretical capacity.^[^ ^1^ ^]^ A relatively low density of sulfur is advantageous for lightweight or high specific energy devices.^[^ ^2^ ^]^ Moreover, the active material sulfur is inexpensive and environmentally friendly.^[^ ^3^ ^]^ However, at present, the high theoretical capacity of Li‐S batteries has not been sufficiently achieved. The main reason is that the sulfur conversion to form lithium sulfide, Li_2_S via an intermediate of lithium polysulfides (LiPSs) takes place in multiple phases.^[^ ^4^ ^]^ Specifically, the conversion from electrolyte‐soluble LiPSs to insoluble Li_2_S upon discharge is sluggish with a high energy barrier due to a large decrease in system entropy.^[^ ^5^, ^6^ ^]^ Moreover, the growth of Li_2_S by electrochemical reduction on the cathode substrate is uncontrollable, often resulting in ionically/electrically passivating the substrate to inhibit full utilization of sulfur.^[^ ^7^, ^8^ ^]^

Much effort has been devoted to introducing advanced materials as cathode substrates.^[^ ^9^, ^10^ ^]^ The metal compound traps LiPSs with high chemical affinity and also serves as a mediator to promote the conversion of LiPSs.^[^ ^11^ ^]^ Oxides have been widely applied because of their facile synthesis into various morphologies and high surface polarity.^[^ ^12^, ^13^ ^]^ The oxide also chemically immobilizes LiPSs by thiosulfate‐polythionate conversion.^[^ ^14^ ^]^ In addition to oxides, sulfides, nitrides, and phosphides have been demonstrated due to their high electrical conductivity as well as their polar surfaces.^[^ ^15^, ^16^, ^17^, ^18^, ^19^ ^]^ Recently, metal compound composites have been introduced to regulate multi‐step sulfur conversion.^[^ ^20^, ^21^ ^]^ For example, the composite substrate of MoS_2_ and MoN has been introduced to enhance the adsorption and conversion of LiPSs, respectively.^[^ ^20^ ^]^ The composites of iron phthalocyanine and octafluoronaphthalene have been introduced into cathode substrates to facilitate the conversion of higher and lower‐order LiPSs, respectively.^[^ ^21^ ^]^ They have been particularly successful in achieving cell stability by promoting the adsorption/conversion of LiPSs. However, the cathode substrate, which also controls the growth of Li_2_S, remains a challenge.

The control of Li_2_S growth has rather been achieved by controlling the donicity of the electrolyte. This approach prevented the passivation of the electrode surface by the growth of Li_2_S, thus achieving high sulfur utilization.^[^ ^22^, ^23^ ^]^ Specifically, a high donicity electrolyte such as dimethyl sulfoxide (DMSO) induced partial solvation of Li_2_S, thereby inhibiting the film‐like growth of Li_2_S.^[^ ^22^, ^23^ ^]^ This effect has also been demonstrated in electrolytes containing high donicity salts such as lithium bromide and lithium nitrate.^[^ ^23^ ^]^ Recently, the application of 1,3‐dimethyl‐2‐imidazolidinone (DMI) has been confirmed to promote the formation of particulate Li_2_S by activating the pathway to generate Li_2_S via sulfide radicals.^[^ ^24^ ^]^ This result exhibited a high discharge capacity of 1595 mAh g^–1^, corresponding to 95% sulfur utilization. Despite these achievements, the reforming of the electrolyte still has to resolve issues such as corrosion of Li anodes and interactions with metal compound substrates.^[^ ^24^, ^25^ ^]^ Therefore, there is still great demand for a substrate capable of controlling Li_2_S growth for the complete and reversible utilization of sulfur.

**Figure 1:**
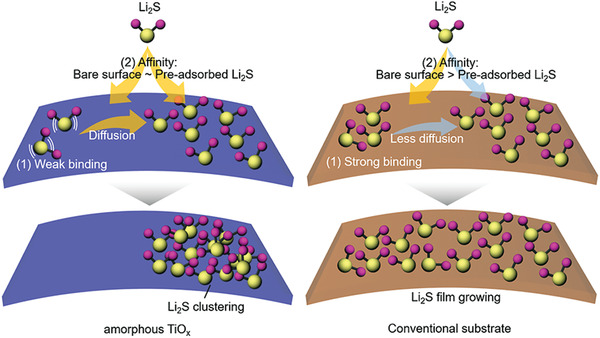
Scheme depicting the growth mechanism of particulate Li_2_S on a a‐TiO_x_ substrate. (Left) On the a‐TiO_x_, the affinity for Li_2_S is relatively weak, and the affinity between the bare surface and the adsorbed Li_2_S surface is similar. This case favors the particulate growth of Li_2_S by diffusion and clustering of Li_2_S. (Right) TiO_2_ has a strong affinity for Li_2_S, and this affinity is stronger than the surface on which Li_2_S is adsorbed. This induces a film‐like growth of Li_2_S on the surface.

To tackle this issue, we explored an amorphous titanium suboxide layer‐coated substrate as a cathode substrate. Amorphous metal oxides have been widely applied as catalysts due to their high activity originating from abundant defective sites.^[^ ^26^, ^27^, ^28^, ^29^, ^30^, ^31^ ^]^ In addition, in Li‐S cell application, the amorphous metal oxide showed strong adsorption to LiPS molecules. Metal compound‐coated substrates that exhibit strong interactions with LiPSs often exhibit a strong affinity for Li_2_S as well. Cui and co‐workers found that the affinity for sulfide species on polar substrates consisted of chemical bonding and van der Waals forces and estimated a stronger affinity for Li_2_S than long‐chain LiPSs by strong chemical bonding. Wang and co‐workers have confirmed that the NiMoO_4_ surface, which has a strong affinity with LiPSs, exhibits strong binding energy to Li_2_S.^[^ ^32^, ^33^ ^]^ We presume that on such a substrate, Li_2_S is less diffusive and the film‐like Li_2_S overlying the substrate grows (see **Figure** **1**). A substrate with a strong affinity for LiPSs but a weak affinity for Li_2_S can induce the growth of Li_2_S particles.

We confirm by DFT calculation that the amorphous titanium suboxide (a‐TiO_x_) surface exhibits relatively weak binding to Li_2_S despite strong binding to LiPSs; on the TiO_2_ surface for comparison, the stronger binding energy is calculated for Li_2_S than for LiPSs under the same conditions (see Figure 1). Moreover, on the a‐TiO_x_ surface, the affinity for Li_2_S is similar between the Li_2_S‐adsorbed surface and the bare surface; TiO_2_ has a strong affinity for bare surfaces. These results present favorable clustering for Li_2_S on a‐TiO_x_ and growth of Li_2_S covering the bare surface of TiO_2_. We confirm the high discharge capacity is accompanying the growth of particulate Li_2_S on a‐TiO_x_ by chronoamperometry; film‐like Li_2_S is observed on TiO_2_. With the strong adsorption of LiPSs and also the regulation of Li_2_S growth, a‐TiO_x_‐containing cathode Li–S batteries achieve high sulfur utilization. We achieve high sulfur utilization of up to 90% at 0.2 C. At the ultra‐high rate of 10 C, a high specific capacity of 850 mAh g^–1^ with a capacity loss of 0.084% per cycle up to 200 cycles is achieved. At a high sulfur load of 7.5 mg S cm^–2^, a practical areal capacity of 10.1 mAh cm^–2^ is also achieved.

## 2 Results and Discussion

### 2.1 Fabrication of a‐TiO_x_/CNTF

**Figure 2:**
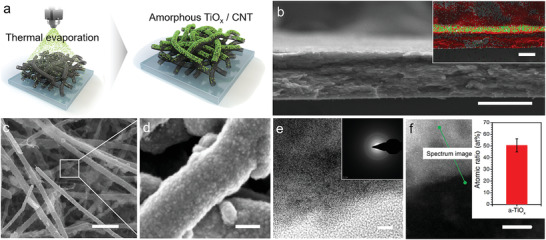
a) Schematic diagram of a‐TiO_x_/CNTF production. b) Cross‐sectional SEM image of a‐TiO_x_/CNTF (scale bar: 100 µm) and its elemental mapping image (inset image, scale bar: 100 µm). c) Low and d) high magnification SEM images of a‐TiO_x_/CNTF (scale bar: 100 µm for c, 100 nm for d). e) High‐resolution TEM image of a‐TiO_x_ and electron diffraction image of the selected area of a‐TiO_x_ (scale bar: 5 nm). f) HAADF‐STEM image of a‐TiO_x_ with a profiling line. The inset is the atomic ratio of Ti/O by EELS.

We obtain a‐TiO_x_ by thermal evaporation from a source of titanium monoxide (TiO). Previously, titanium suboxide was often obtained by catalytic reduction of crystalline TiO_2_ in a high‐temperature hydrogen atmosphere.^[^ ^34^, ^35^, ^36^ ^]^ Compared to this process, thermal evaporation facilitates the preparation of titanium suboxide (see Figure S1, Supporting Information). a‐TiO_x_ is coated on the CNT‐assembled film (CNTF) and applied as a cathode substrate, as described in **Figure** **2**a. The a‐TiO_x_ is mainly coated on the top region of the CNTF because of the directional deposition in a high vacuum (Figure 2b, and S2, Supporting Information). Due to the porous structure of CNTF, a‐TiO_x_ is coated on several layers of CNTs on the surface (Figure 2c).

The a‐TiO_x_ layer consists of particles with sizes on the order of 100 nm (Figure 2d and Figure S2, Supporting Information). The HR‐TEM images and SAED patterns of a‐TiO_x_ reveal an amorphous structure that does not exhibit long‐range order (Figure 2e). During the thermal deposition, the temperature of the CNTF substrate is maintained at room temperature to achieve rapid thermal quenching, whereby an amorphous phase is obtained.

A high‐angle annular dark‐field scanning transmission electron microscope (HAADF‐STEM) image (Drift‐corrected) of a‐TiO_x_ with a profiling line is displayed in Figure 2f. The inset shows the atomic ratio of Ti and O by electron energy loss spectroscopy (EELS). The EELS analysis confirms that the O/Ti ratio in a‐TiO_x_ ranges from 0.8 to 1.2. We determine that a‐TiO_x_ has the composition of TiO. In the phase diagram for the Ti/O ratio, it is confirmed that monoclinic TiO exists as an independent phase at the ratio ≈1.^[^ ^37^, ^38^ ^]^

### 2.2 Exploring Lithium Polysulfide Adsorption and Lithium Sulfide Growth on a‐TiO_x_

First, we analyze the adsorption of LiPS on a‐TiO_x_/CNTF. For comparison, TiO_2_‐coated CNTF (TiO_2_/CNTF) is prepared. The TiO_2_/CNTF sample is obtained by annealing a‐TiO_x_/CNTF at 480 °C in an air atmosphere (Note S1, Supporting Information); this temperature is inert to CNTF. The preparation of the control sample by this process minimizes the morphological difference from the a‐TiO_x_/CNTF sample. The XRD and Raman spectra of the TiO_2_/CNTF confirm crystalline TiO_2_ (Figure S3, Supporting Information).

**Figure 3:**
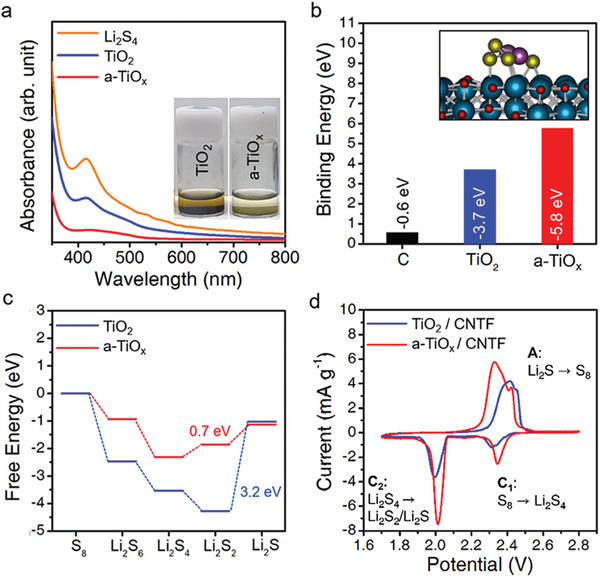
a) UV–vis spectra of electrolytes containing Li_2_S_4_, and UV–vis spectra of Li_2_S_4_ electrolytes after 4 h immersion of a‐TiO_x_/CNTF and TiO_2_/CNTF. Inset is a digital camera image of an electrolyte containing a‐TiO_x_/CNTF and TiO_2_/CNTF, respectively. b) Binding energy of Li_2_S_4_ on carbon, TiO_2,_ and a‐TiO_x_, respectively, by DFT calculation. Inset is an energy‐optimized atomic configuration image of Li_2_S_4_ adsorbed on a‐TiO_x_. Titanium monoxide (TiO) is applied as an atomic model for TiO_x_. c) The individual free energy profiles for the discharge process on a‐TiO_x_ and TiO_2_. d) Cyclic voltammetry curves for a‐TiO_x_/CNTF and TiO_2_/CNTF electrodes at a scan rate of 0.1 mV s^–1^.

The inset shows digital camera images of a‐TiO_x_/CNTF and TiO_2_/CNTF samples after 3 h adsorption immersed in an electrolyte containing Li_2_S_4_. Compared to the TiO_2_ sample, the a‐TiO_x_ sample shows an almost transparent solution (**Figure** **3**a). In Figure 3a, the UV–vis spectra of the electrolyte solutions of each sample after the adsorption are compared; the peak at 421 nm is attributed to the absorption by Li_2_S_4_.^[^ ^39^ ^]^ The absorption peak of a‐TiO_x_/CNTF is significantly lower than that of TiO_2_/CNTF. The a‐TiO_x_ substrate has a higher adsorption capacity for Li_2_S_4_ compared to TiO_2_.

The strong affinity of a‐TiO_x_ for LiPSs can be attributed to the strong Lewis acid‐base interaction due to the vacant d‐orbitals of a‐TiO_x_.^[^ ^40^, ^41^ ^]^ We evaluate the affinity by DFT‐based binding energy calculations. The binding energy is obtained by *E*_total_ – *E*_ads_ – *E*_surf_, where *E*_total_ is the total energy of the absorbed system, *E*_ads_ is the energy of the optimized Li_2_S_6_ in a vacuum, and *E*_surf_ is the energy of the optimized bare surface.^[^ ^42^ ^]^ As an atomic model for a‐TiO_x_, amorphous titanium monoxide (TiO) is constructed using the melt quenching technique.^[^ ^43^, ^44^ ^]^ Specifically, a crystalline model obtained using ab initio molecular dynamics (AIMD) was diffused at 6000 K and then quenched at 300 K.^[^ ^45^, ^46^ ^]^ This melting and quenching technique through AIMD has been previously applied to construct an amorphous interface of Sb_2_Te_3_. In Figure 3b, the binding energies of Li_2_S_4_ on carbon, TiO_2,_ and a‐TiO_x_ are compared. The carbon surface exhibits weak adsorption to Li_2_S_4_, with a binding energy of only −0.6 eV. The binding energy on the a‐TiO_x_ surface is 56% higher than that on TiO_2_. We also analyze the average distance between the S atoms and the atoms on the substrate surface, confirming that the interatomic distance is 85% shorter on a‐TiO_x_ than on TiO_2_. These results indicate stronger bonding of Li_2_S_4_ on a‐TiO_x_ and correspond well with the results of adsorption experiments.

Second, we compare the kinetics of sulfur conversion on both substrates. Through DFT calculations, we compare the Gibbs free energy profiles for the discharge process on the two substrates (Figure 3c and see Figure S4, Supporting Information). Both substrates exhibit spontaneous exothermic reactions in the process of sulfur to Li_2_S_2_. However, an energy barrier is observed in the formation of Li_2_S, which has been reported as a rate‐determining step.^[^ ^47^, ^48^ ^]^ Compared to TiO_2_, the a‐TiO_x_ substrate exhibits much lower barrier energy. The barrier energies for a‐TiO_x_ and TiO_2_ are 0.7 and 3.2 eV, respectively. This result indicates a higher rate of sulfur conversion on the a‐TiO_x_ substrate.

Compared with TiO_2_, the enhanced sulfur conversion of a‐TiO_x_ is also confirmed by the response of the reaction peak in the cyclic voltammetry. Figure 3d shows CV curves for a‐TiO_x_/CNTF and TiO_2_/CNTF cathode substrates in LiPS‐containing electrolytes. The current profile shows two reduction peaks and one oxidation peak. Each reduction peak represents the conversion of elemental sulfur to higher LiPSs (Li_2_S_x_, x = 4,6) and lower LiPSs (Li_2_S_x_, x = 1,2), respectively.^[^ ^49^ ^]^ The oxidation sweep shows one peak corresponding to the conversion of lower LiPSs to sulfur. Compared with the TiO_2_/CNTF, the a‐TiO_x_/CNTF shows a higher current at a higher voltage for both reduction peaks and a clearer current peak in the oxidation sweep.^[^ ^16^, ^50^ ^]^ This clearly indicates a faster sulfur conversion reaction on the a‐TiO_x_ surface.

**Figure 4:**
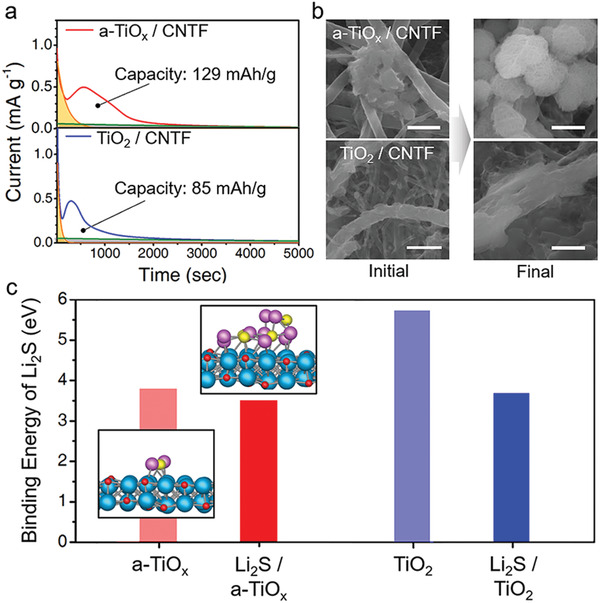
a) Potentiostatic discharge (0.5 m Li_2_S_6_, 2.05 V) curves forming Li_2_S in a‐TiO_x_/CNTF and TiO_2_/CNTF cathodes. b) SEM images of Li_2_S grown on a‐TiO_x_ and TiO_2_ layers of a‐TiO_x_/CNTF and TiO_2_/CNTF cathode substrates, respectively, at the beginning of discharge and after full discharge. (scale bar: 500 nm) c) Binding energy for adsorption of Li_2_S on a‐TiO_x_ and TiO_2_ surfaces, respectively. Binding energy to adsorb Li_2_S to each surface on which four Li_2_S molecules have been pre‐adsorbed. Titanium monoxide (TiO) is applied as an atomic model for TiO_x_.

In the CV analysis, the a‐TiO_x_ substrate shows excellent kinetics for the sluggish formation of Li_2_S during discharge. We analyze the capacity for Li_2_S_4_ to Li_2_S conversion and the morphology of Li_2_S through chronoamperometry. The measurement is achieved by applying a voltage of 2.05 V to the TiO_2_/CNTF or a‐TiO_x_/CNTF substrate in an electrolyte containing Li_2_S_4_. The discharge profile and the growth morphology of Li_2_S after discharge on each substrate are presented in **Figures** **4**a,b, respectively. The a‐TiO_x_/CNTF cathode cell shows a longer discharge time than the TiO_2_/CNTF cell. The calculated capacity based on the integral area of the discharge curve is 129 mAh g^–1^ for the a‐TiO_x_/CNTF, which is 53% larger than that of the TiO_2_/CNTF. For the bare CNTF electrode, only a capacity of 62 mAh g^–1^ was obtained. (see Note S2, Supporting Information)

Note that the formation of particulate Li_2_S is observed on the a‐TiO_x_/CNTF surface (Figure 4b, see also Note S2, Supporting Information). It has been reported that such morphological growth does not impede the transport of lithium ions/charges, resulting in high sulfur utilization.^[^ ^51^, ^52^ ^]^ The high capacity for Li_2_S_4_‐to‐Li_2_S conversion in a‐TiO_x_/CNTF is attributed to this particle growth. In contrast, the TiO_2_/CNTF sample exhibits film‐like growth of Li_2_S, which results in poor sulfur utilization. (see Figure S6, Supporting Information). Note the growth of particulate Li_2_S despite the strong adsorption of a‐TiO_x_ to LiPS. On a substrate with strong adsorption capacity, abundant LiPSs are present on the surface, which facilitates the growth of the film‐like Li_2_S with rapid nucleation‐growth; Nazar and co‐workers have observed the formation of Li_2_S films by adsorption of LiPS molecules and surface‐mediated reduction on metallic polar Ti_4_O_7_.^[^ ^53^ ^]^

To analyze the particulate growth on the a‐TiO_x_ surface, the binding energy for Li_2_S on the TiO_2_ and a‐TiO_x_ surfaces is compared by DFT calculation. a‐TiO_x_ shows weak binding corresponding to only 66% of the binding energy on TiO_2_, as shown in Figure 4c. This implies that the reduced Li_2_S molecules diffuse easily on the a‐TiO_x_ substrate, as depicted in Figure 1. Previously, Kim and co‐workers have reported the growth of particulate Li_2_S by diffusion of partially dissolved Li_2_S and also clustering of Li_2_S.^[^ ^52^ ^]^ Moreover, on each substrate, we calculate the binding energy of Li_2_S on the bare surface and on the surface on which Li_2_S is pre‐adsorbed (see Figure 4c). The TiO_2_ surface exhibits stronger binding energy on the bare surface than the Li_2_S‐adsorbed surface. This reveals that the growth covering the bare surface, i.e., a film‐like growth, is thermodynamically favorable on the TiO_2_ surface. In contrast, for the a‐TiO_x_, the binding energies on both surfaces are similar. That is, the clustering of Li_2_S is more favorable on the surface of a‐TiO_x_ than on TiO_2_, as depicted in Figure 1. Considering the effect of the electrolyte solvent to stabilize the sulfide, the clustering between Li_2_S can be further promoted by a similar action to the hydrophobic interaction.^[^ ^52^, ^53^, ^54^ ^]^ Briefly, the DFT calculations reveal favorable surface diffusion of Li_2_S and also clustering between Li_2_S on a‐TiO_x_, supporting the particulate growth observed on a‐TiO_x_ substrates.

### 2.3 Electrochemical Performance of a‐TiO_x_/CNTF Cells

**Figure 5:**
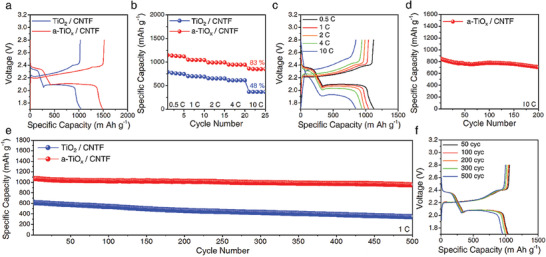
a) Voltage profiles of TiO_2_/CNTF and a‐TiO_x_/CNTF cathode cells at 0.2 C. b) Discharge capacity of TiO_2_/CNTF and a‐TiO_x_/CNTF cathode cells at various current densities from 0.5 C to 10 C. c) Voltage profiles of a‐TiO/CNTF cathode cells at various current densities from 0.5 C to 10 C. d) Cycling performance for a‐TiO_x_/CNTF cathode cells at 10 C. e) Cycling performance for TiO_2_/CNTF and a‐TiO_x_/CNTF cathode cells at 1 C. f) Voltage profiles of a‐TiO_x_/CNTF cathode cells at 1 C.

We prepare a Li‐S battery containing a cathode substrate of sulfur‐loaded a‐TiO_x_/CNTF. The areal loading mass of sulfur is typically 1 mg cm^–2^ (Figure S6, Supporting Information). The charge–discharge profile of a‐TiO_x_/CNTF cell at 0.2 C is shown in **Figure** **5**a. The a‐TiO_x_/CNTF cell delivers a capacity of 1502 mAh g^–1^, corresponding to ≈90% sulfur utilization. This sulfur utilization is superior to recent results for metal compound/carbon cathode substrate cells (Table S1, Supporting Information) and is comparable to the values achieved with controlled growth of Li_2_S in high donicity electrolytes.^[^ ^23^, ^24^, ^52^, ^55^ ^]^ The discharge capacity of the TiO_2_/CNTF cell is 1050 mAh g^–1^, which is only 70% of the capacity of the a‐TiO_x_/CNTF cell.

The discharge capacities of a‐TiO_x_/CNTF and TiO_2_/CNTF cells are recorded with a 20‐fold increase in the C‐rate from 0.5 C to an ultrafast rate of 10 C (Figure 5b). The capacity of the a‐TiO_x_/CNTF cell at 10 C exhibits high retention of 83% of the capacity at 0.5 C, whereas the retention of the TiO_2_/CNTF cell is only 48%. The capacity at 10 C of the a‐TiO_x_/CNTF cell, 850 mAh g^–1^, is superior to the previous results highlighting the high capacity at a high rate (Figure S7, Supporting Information). In Figure 5c, the discharge–charge profile of the a‐TiOx/CNTF cell shows two stable plateaus revealing a reversible sulfur redox reaction even at a high current density of 10 C‐rate. In addition, the a‐TiO_x_/CNTF cell exhibits a reversible charge/discharge cycle even at 10 C, as shown in Figure 5d. At 200 charge/discharge cycles, the specific capacity is 707 mAh g^–1^, maintaining 83% of the initial capacity. The a‐TiOx/CNTF cell shows a stable voltage profile up to 200 cycles even at a current density of 10 C (Figures S8 and S9, Supporting Information).

The cyclic stability of a‐TiO_x_/CNTF cells is evaluated at 1 C, as shown in Figure 5e. During 500 cycles, the a‐TiO_x_/CNTF cell exhibits high stability with a capacity loss of 0.0226% per cycle; the TiO_2_/CNTF cell shows a capacity loss per cycle four times greater. The discharge/charge profile in Figure 5f shows a stable profile for up to 500 cycles (see Figure S9, Supporting Information). The a‐TiO_x_/CNTF cathode cell achieves high reversible capacity, especially at high rates. This is due to the controlled growth of Li_2_S as well as the continuous and complete conversion of LiPSs to Li_2_S on a‐TiO_x_/CNTF substrates; We confirm the formation of particulate Li_2_S observed in the chronoamperometry also in constant current discharge (Figure S10, Supporting Information).

**Figure 6:**
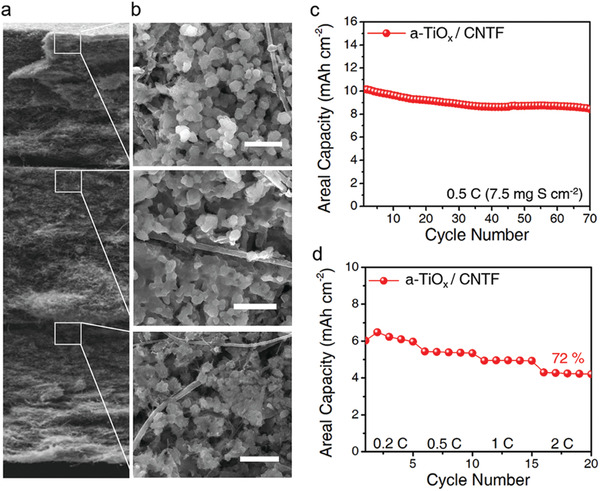
a) Cross‐sectional SEM image of three layers of a‐TiO_x_ / CNTF (scale bar: 50 µm). b) SEM images after full discharge in selected regions of the cathode. Particulate Li_2_S is shown in the a‐TiO_x_ coating layer of each a‐TiO_x_/CNTF (scale bar: 1 µm). c) Cycling performance for a‐TiO_x_/CNTF particle cathodes at 0.5 C with a high sulfur loading of 7.5 mg S cm^–2^. d) Discharge capacities of a‐TiO_x_/CNTF cathode cells at various current densities of 0.2 C–2 C. (Sulfur loading: 5 mg S cm^–2^).

The high areal capacity that requires high sulfur loading cathodes is a crucial metric for practical applications of Li‐S batteries.^[^ ^56^ ^]^ We stacked three a‐TiO_x_/CNTFs to obtain a high sulfur loading electrode of 7.5 mg cm^–2^. The cross‐sectional SEM, shown in **Figure** **6**a, shows a film composed of three layers of a‐TiO_x_/CNTF. The operando EIS analysis of this multilayer a‐TiO_x_/CNTF cathode substrate cell shows low interfacial resistance between layers and also reversible sulfur‐Li_2_S conversion over this substrate maintained during charge/discharge (Figure S11, Supporting Information). We also confirm the growth of particulate Li_2_S in the multilayer a‐TiO_x_/CNTF; The SEM image shown in Figure 6b shows particulate Li_2_S in the a‐TiO_x_‐coated region at full discharge. The areal capacity of the multilayer a‐TiO_x_/CNTF cell evaluated at 0.5 C delivers 10.1 mAh cm^–2^ at initial discharge and 8.5 mAh cm^–2^ at 70 cycles (Figure 6c and S9, Supporting Information). This value exceeds the typical areal capacity range of 2 – 4 mAh cm^–2^ for conventional lithium‐ion batteries.^[^ ^57^, ^58^ ^]^

The areal capacity is assessed at an increasing C‐rate from 0.5 C to 2 C (Figure 6d). The multilayer a‐TiO_x_/CNTF cell delivers a high capacity of 4.3 mAh cm^–2^ even under high current density conditions of 2 C (see Figure S12, Supporting Information). We use the Ragone plot to compare cell performance from a practical perspective of various composite substrate cells and a‐TiO_x_/CNTF cells. The a‐TiO_x_/CNTF cell achieves high energy density and power density simultaneously (Figure S13, Supporting Information).

## 3 Conclusion

A cathode substrate that regulates the multiple steps of the sulfur conversion reaction is urgently needed for practical Li‐S batteries. We present an a‐TiO_x_ that strongly adsorbs LiPS and also controls the growth of Li_2_S. We prepare a‐TiO_x_ by thermal evaporation and apply an a‐TiO_x_‐coated CNTF as a cathode substrate. First, the a‐TiO_x_ shows a high adsorption capacity for LiPS, which is also confirmed by the binding energy obtained by the DFT calculation. Compared to TiO_2_, the a‐TiO_x_ substrate shows an ≈56% improvement in the adsorption amount of LiPS. In addition, the a‐TiO_x_/CNTF substrate exhibits excellent kinetics for sulfur conversion. In the CV measurements, the a‐TiO_x_/CNTF shows a reduction current that is twice that of TiO_2_ for the sluggish reaction of LiPS‐to‐Li_2_S. We confirm that the excellent kinetics in a‐TiO_x_/CNTF is related to the growth of particulate Li_2_S on the a‐TiO_x_ layer surface. On the TiO_2_, a film‐like Li_2_S is formed in contrast. We confirm that the formation of particulate Li_2_S on a‐TiO_x_ despite its high adsorption capacity for LiPS is due to the relatively weak affinity for Li_2_S on a‐TiO_x_. In addition, the DFT calculation confirms that the binding energy for Li_2_S on the Li_2_S‐adsorbed a‐TiO_x_ surface and the bare a‐TiO_x_ surface is similar. Through these results, we present that thermodynamically favorable diffusion and clustering of Li_2_S induces the growth of particulate Li_2_S on the a‐TiOx substrate. Due to this dual function of a‐TiO_x_ in sulfur conversion, the a‐TiO_x_/CNTF cathode‐based Li‐S battery achieves high sulfur utilization of 90%. The a‐TiO_x_/CNTF cathode cell also exhibits high stability even at an ultra‐high rate of 10 C with a discharge capacity of 850 mAh g^–1^ and a capacity reduction of 0.0226% per cycle up to 500 cycles. High sulfur loadings for practical Li‐S batteries are readily achieved by stacking a‐TiO_x_/CNTFs. We achieve an areal capacity of 10.1 mAh cm^–2^ in a multilayer a‐TiO_x_/CNTF cathode cell with a sulfur loading of 7.5 mg cm^–2^; this greatly exceeds the areal capacity of conventional Li‐ion batteries. Our simple processing and high‐functionality cathode substrate will speed up the practical use of Li‐S batteries.

## 4 Experimental Section

### Preparation of a‐TiO_x_/CNTF

CNT films were produced by vacuum filtration of multiwall CNT dispersions. a‐TiO_x_ was coated by thermal evaporation of titanium monoxide (THIFINE, 99.9%). During the coating, the oxidation proceeds to acquire titanium suboxide (TiO_x_). Thermal evaporation was performed at a pressure of 10^–5^ Torr, where the deposition rate was 0.5 Å s^–1^. The substrate holder was kept at room temperature. The preparation of TiO_2_/CNTF for comparison was obtained by annealing a‐TiO_x_/CNTF at 480 °C for 30 min in an air atmosphere.

### Electrochemical Characterization

To obtain a CV curve and a galvanostatic charge/discharge profile, an electrolyte solution in which 1 M bis (trifluoromethane) sulfonimide lithium salt (LiTFSI, Sigma‐Aldrich) and 0.2 m lithium nitrate (LiNO_3_, Alfa Aesar) was dissolved in a 1:1 v/v% of 1,3‐dioxolane (DOL, Sigma‐Aldrich)/dimethylethane (DME, Sigma–Aldrich) was applied. The loading of sulfur on the cathode substrate was prepared by drop‐casting sulfur‐dissolved carbon disulfide. The potentiostatic discharge experiment uses a polysulfide catholyte. The catholyte was obtained by mixing sulfur and Li_2_S in the electrolyte at a molar ratio of 5:1. The cell was prepared in a coin‐cell type using a cathode, a Li metal (1 mm thick) anode, and a polypropylene membrane separator (25 µm, Celgard). Cells were assembled in a controlled humidity environment (<0.3%). The ratio of electrolyte/sulfur (E/S) is 7. The low sulfur loading condition applies E/S = 15. The specific capacity was calculated based on the sulfur mass in the cathode. Cell performance was tested using a Maccor 4300 system. The test temperature was 25 °C. No pre‐cycling was performed. The theoretical capacity utilizes 1675 mAh g^–1^; the specific capacity was calculated based on the average CE value of all cell results being greater than 99%.

### Calculation Details

All calculations were performed using the Quantum ESPRESSO package based on density functional theory. A generalized gradient approximation (GGA) with a Perde‐Burke‐Ernzerhof (PBE) exchange‐correlation function was used. A plane‐wave basis set with a cutoff energy of 408 eV was applied to the calculations. The set of k‐points selected by the Monkhorst‐Pack scheme was used to obtain the consolidation of the Brillouin zone. For structural optimization, all ions were relaxed up to a maximum force of 0.02 eV Å^–1^.

### Material and Structural Characterization

The surface morphology was investigated by field emission scanning electron microscopy (FE‐SEM, Carl Zeiss, Supra 55 VP) and transmission electron microscopy (TEM, JEM‐3010, JEOL). Elemental mapping was obtained using energy dispersive spectroscopy (EDS, BRUKER, XFlash Detector 4010). X‐ray diffraction (XRD) spectra were collected on a Davinci D8 Advance diffractometer using Cu‐K*α* radiation. (The scanning was in the range of 20°– 80° at a scan rate of 0.05° s^–1^.) X‐ray photoelectron spectroscopy (XPS) was performed using a Leybold spectrometer with an Al K*α* monochromatic beam (1486.6 eV) (150 W input power, ESCALAB250 XPS system, Theta Probe XPS system). Raman spectra were acquired using a Horiba Jobin Yvon LabRAM HR equipped with an air‐cooled Ar‐ion laser operating at 514 nm. Measurements of HAADF‐STEM and EELS were performed using a spherical aberration‐corrected JEM ARM‐200F microscope (Cold FEG Type, JEOL) equipped with an EELS detector (965 GIF Quantum ER, GATAN). In the HAADF‐STEM images, the point‐to‐point resolution was ≈80 pm after Cs correction, and the angular range of the annular detector was 68–280 mrad. For the EELS, the energy dissipation was set to 0.25 eV ch^–1^, and the full‐width at half‐maximum of the zero‐loss peak in vacuum was 1.0 eV. The convergence and collection semi‐angles were 19 and 26 mrad, respectively. Digital microscope software (GMS 3.3, GATAN) was used for image recording and processing.

## Conflict of Interest

The authors declare no conflict of interest.

## Supporting information

Supporting Information

## Data Availability

The data that support the findings of this study are available from the corresponding author upon reasonable request.
