# Supplementary material for: Discovery of Dual‐Functional Amorphous Titanium Suboxide to Promote Polysulfide Adsorption and Regulate Sulfide Growth in Li–S Batteries
Source: Adv Sci (Weinh). 2022 Jun 5;9(22):2200958. doi: 10.1002/advs.202200958 (PMC9353452; doi:10.1002/advs.202200958)
Supplement: Supplementary file 1 — Supporting Information [file ADVS-9-2200958-s001.pdf]

ADVANCED SCIENCE
Open Access

Supporting Information

for *Adv. Sci.*, DOI 10.1002/advs.202200958

# Discovery of Dual-Functional Amorphous Titanium Suboxide to Promote Polysulfide Adsorption and Regulate Sulfide Growth in Li–S Batteries

*Donghee Gueon, Jisu Yoon, Jinhan Cho and Jun Hyuk Moon**

# Supporting Information

## Discovery of dual-functional amorphous titanium suboxide to promote polysulfide adsorption and regulate sulfide growth in Li-S batteries

*Donghee Gueon[1], Jisu Yoon[1], Jinhan Cho[2,3] and Jun Hyuk Moon[1,*]*

[1] Department of Chemical and Biomolecular Engineering, Institute of Emergent Materials, Sogang University, Baekbeom-ro 35, Mapo-gu, Seoul, 04107, Republic of Korea
[2] Department of Chemical and Biological Engineering Korea University 145 Anam-ro, Seongbuk-gu, Seoul 02841, Republic of Korea
[3] KU-KIST Graduate School of Converging Science and Technology Korea University 145 Anam-ro, Seongbuk-gu, Seoul 02841, Republic of Korea

**Corresponding Author**
[1]Jun Hyuk Moon, E-mail: junhyuk@sogang.ac.kr

**Supplementary Information #1**

We compared the XPS analysis of the CNTF before and after the heat treatment at 480 °C. We deconvolute the spectrum into peaks corresponding to $H_2O$, C-O-H, C-O-C and C=O, and calculate the area of each peak. The peak area ratio of the bare CNT sample is $H_2O$ : C-O-H : C-O-C : C=O = 4.7 : 27 : 35.7 : 31.8, and the peak area ratio of the annealed-CNT sample is $H_2O$ : C-O-H : C-O-C : C=O = 5.5 : 31.4 : 35.7 : 28.2.

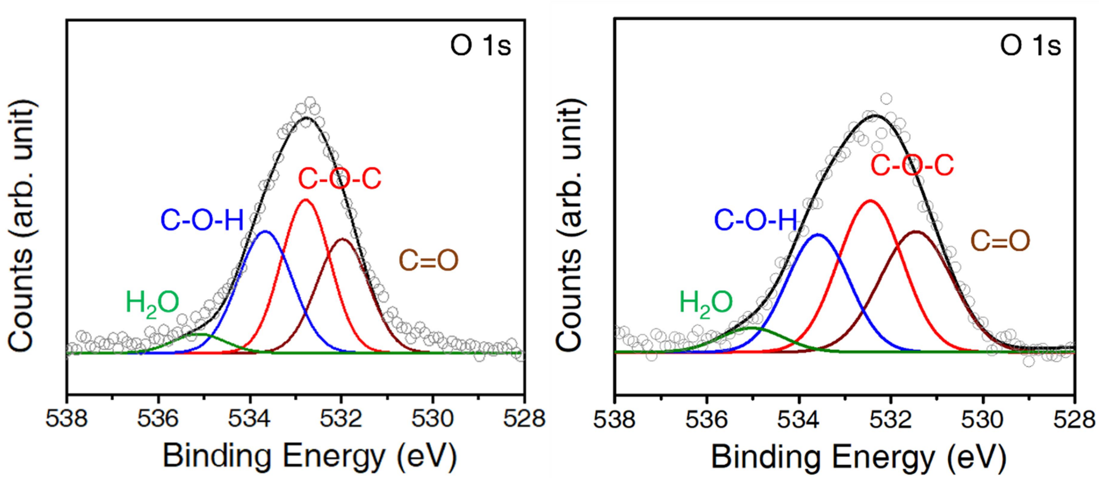


Figure. O 1s XPS spectra on CNTF (left) and annealed CNTF (right).

The composition ratio of the surface oxygenate groups in the two samples is similar; the heat treatment does not cause significant changes in surface functional groups.

**Supplementary Information #2**

A potentiostatic discharge is performed under the condition that a constant potential sufficient to form $Li_2S$ in the $Li_2S_4$-containing electrolyte is applied.

The growth of particulate $Li_2S$ is observed on the a-$TiO_x$-coated side.

However, we observed film-like growth on the CNTF surface opposite to the coating direction on the a-$TiO_x$/CNTF electrode (see the SEM images of the top panel). This growth morphology is similar to that of the bare CNTF electrode shown in the bottom panel. Note that, although the two substrates are the same, the growth rate of the bare CNTF region of the a-$TiO_x$/CNTF electrode is slower than that of the bare CNTF. This confirms the dominant growth in the a-$TiO_x$ coated region in the a-$TiO_x$/CNTF cathode substrate. This shows that although a-$TiO_x$ has a limited coating on the Top region of the CNTF, sufficient sulfur-conversion can be achieved, and a concomitant high discharge capacity can be expected.

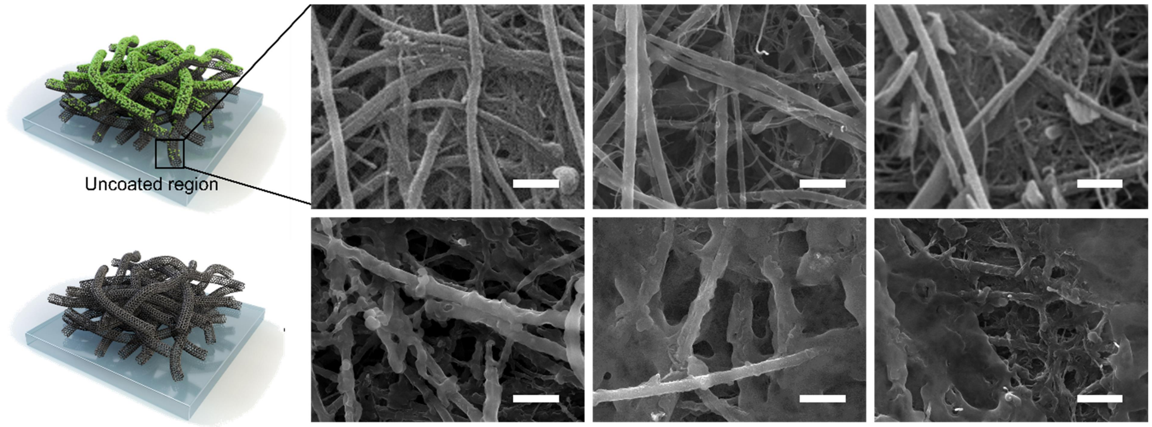


Figure. (Top panel) SEM images of the growth morphology of $Li_2S$ during discharge of the a-$TiO_x$ barely coated region in a-$TiO_x$/CNTF. (Bottom panel) SEM images showing the growth of $Li_2S$ during discharge in bare CNTF. The image on the Right shows a more complete discharge. (scale bar: 1 μm)

The figure below compares the morphologies of $Li_2S$ on a-$TiO_x$/CNTF and $TiO_2$/CNTF. In contrast to the a-$TiO_x$/CNTF substrate, a thick $Li_2S$ film was observed on the $TiO_2$/CNTF substrate.

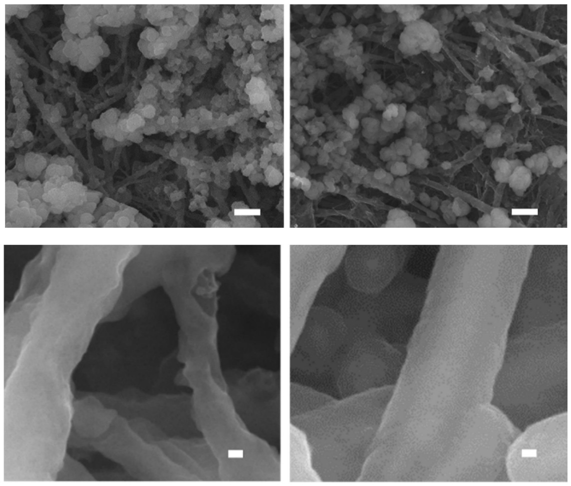

Figure. (top panel) Low magnification SEM images showing particulate $Li_2S$ on the a-$TiO_x$ surface. (scale bar: 1 μm) (bottom panel) High magnification SEM images of $TiO_2$/CNTF electrodes after potentiostatic discharge, respectively. (scale bar: 100 nm) The Left and Right images are images at initial discharge and after full discharge, respectively. A thick $Li_2S$ film was observed on the $TiO_2$/CNTF substrate.

We use the normalized I-t curve to determine the growth mode of $Li_2S$ on a-$TiO_x$/CNTF and $TiO_2$/CNTF. In the figure below, the dotted lines represent 2D and 3D diffusion-growth of lithium sulfides based on classical electrodeposition theory. We utilize Bewick, Fleischmann, and Thirsk models (BFT models) and Scharifker-Hills models (SH models) as models for 2D and 3D growth, respectively. In these models, progressive nucleation (designated "P") and instantaneous nucleation (designated "I") are distinguished. [Unraveling the Dual Functionality of High-Donor-Number Anion in Lean-Electrolyte Lithium-Sulfur Batteries, *Adv. Energy Mater*. 2020]

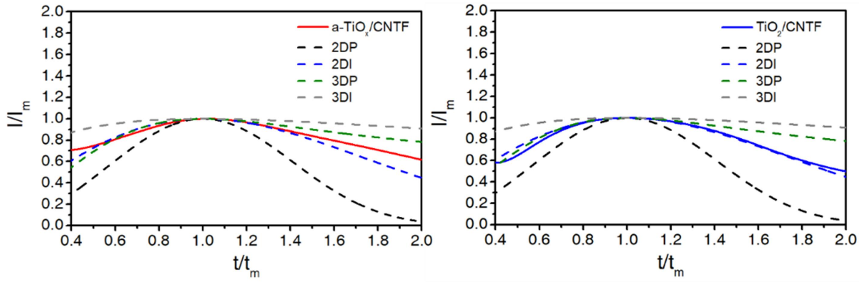


Figure. Normalized I-t curves for nucleation-growth of $Li_2S$ in a-$TiO_x$/CNTF and $TiO_2$/CNTF.

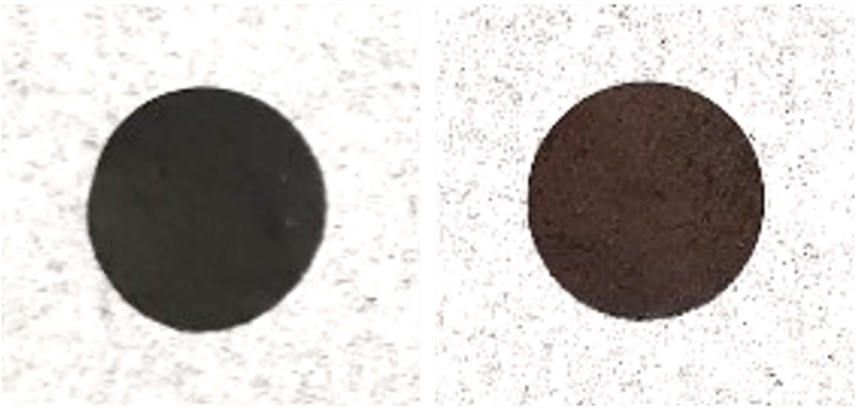

**Figure S1.** Digital camera images (Left) before and (Right) after the thermal deposition on CNT film samples. The coated surface appears brown, which is in good agreement with the results reported for titanium monoxide in the literature.[1]

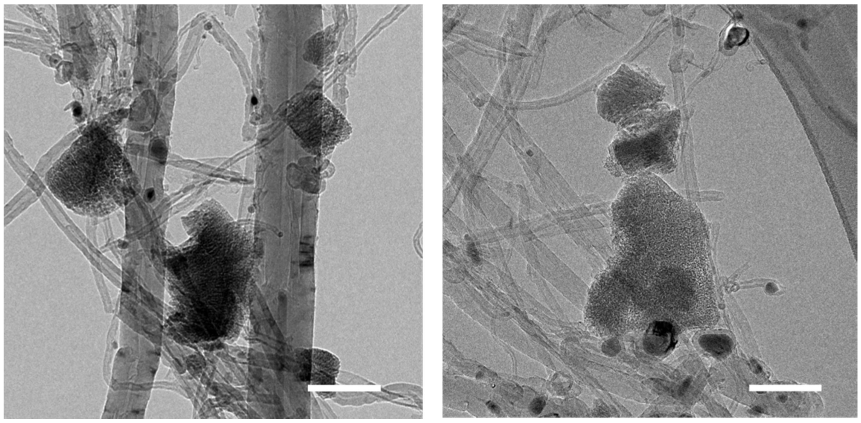

**Figure S2.** TEM image of the a-$TiO_x$ coated on CNT film. (scale bar: 100 nm)

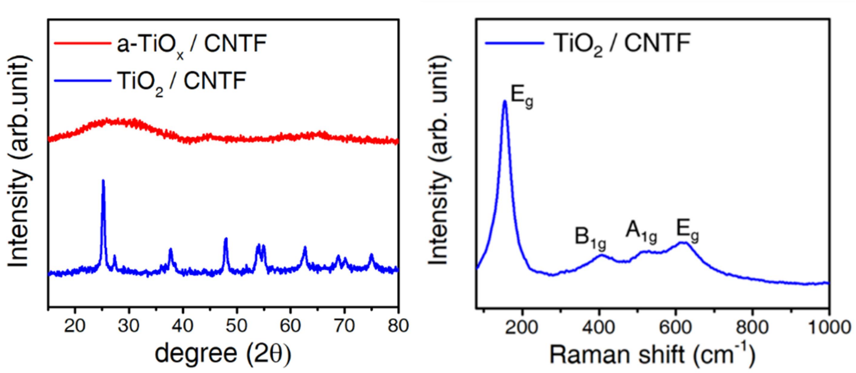


**Figure S3.** (left) XRD spectra of a-$TiO_x$/CNTF and $TiO_2$/CNTF. The strong diffraction peaks at 25° and 48° indicate anatase crystalline phase. (right) Raman spectrum of $TiO_2$/CNTF. The bands 198, 398, and 640 $cm^{-1}$ are typical of the anatase crystal phase. These results confirm that the sample obtained by sintering a-$TiO_x$/CNTF corresponds to the crystalline $TiO_2$/CNTF.

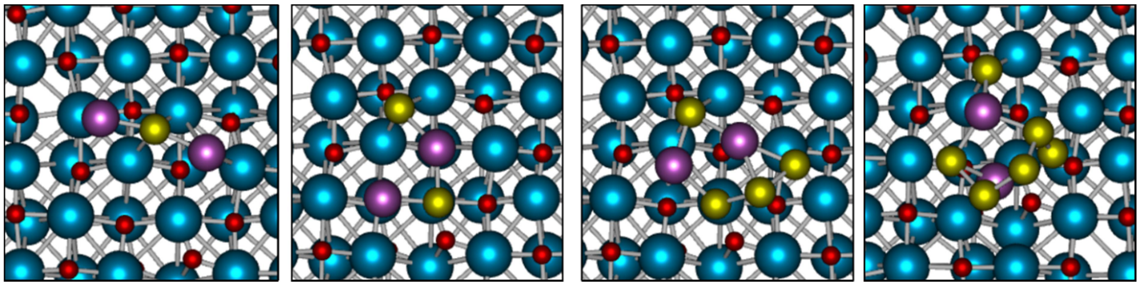

**Figure S4.** Optimized adsorption configuration images of each $Li_2S$, $Li_2S_2$, $Li_2S_4$, or $Li_2S_6$ molecule on the a-$TiO_x$ surface for Gibbs energy calculation. Titanium monoxide (TiO) was applied as an atomic model for $TiO_x$. Subsequently, the melting and quenching technique through AIMD was applied to construct an amorphous phase.

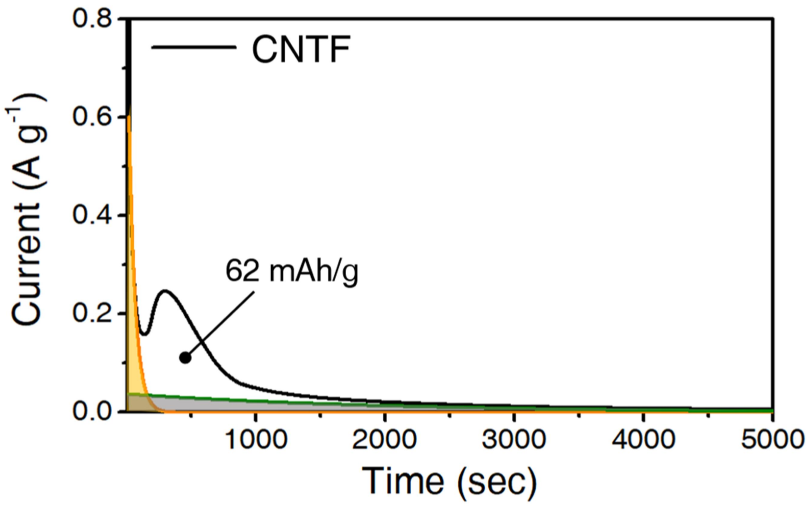


**Figure S5.** Potentiostatic discharge curve for the bare CNTF cathode. The integral value of the white region under the curve represents the capacity to form $Li_2S$ from $Li_2S_4$.

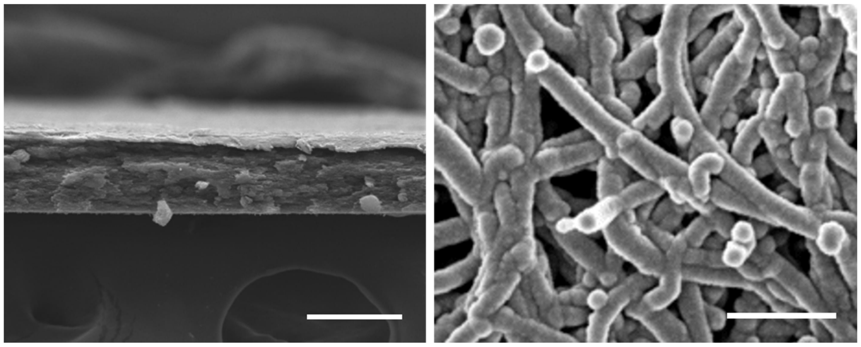

**Figure S6.** Cross-sectional and surface SEM image of sulfur-loaded a-$TiO_x$/CNTF. (scale bar: (Left) 100 μm and (Right) 500 nm).

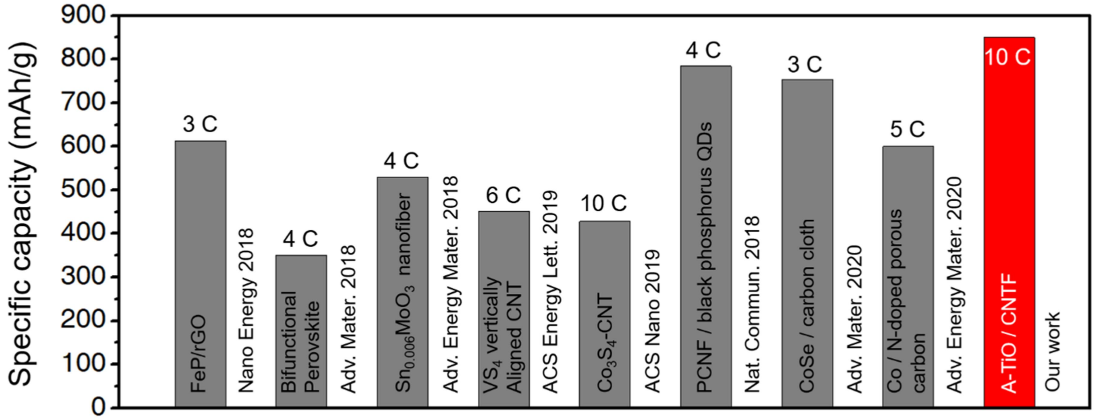


**Figure S7.** Comparison of the capacity of a-$TiO_x$/CNTF electrode cell and recently reported metal compound/carbon composite electrode cell under high C-rate condition.[2-9]

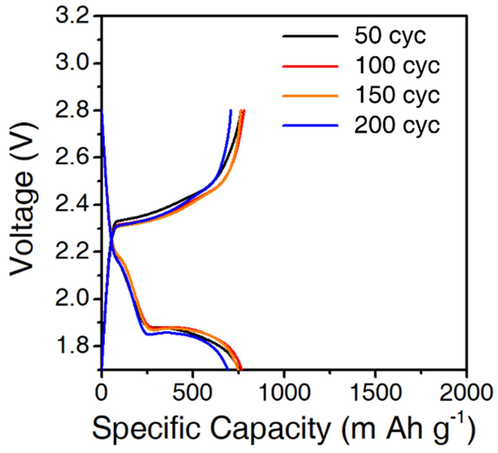


**Figure S8.** Voltage profiles of a-$TiO_x$/CNTF cathode cells at 10 C.

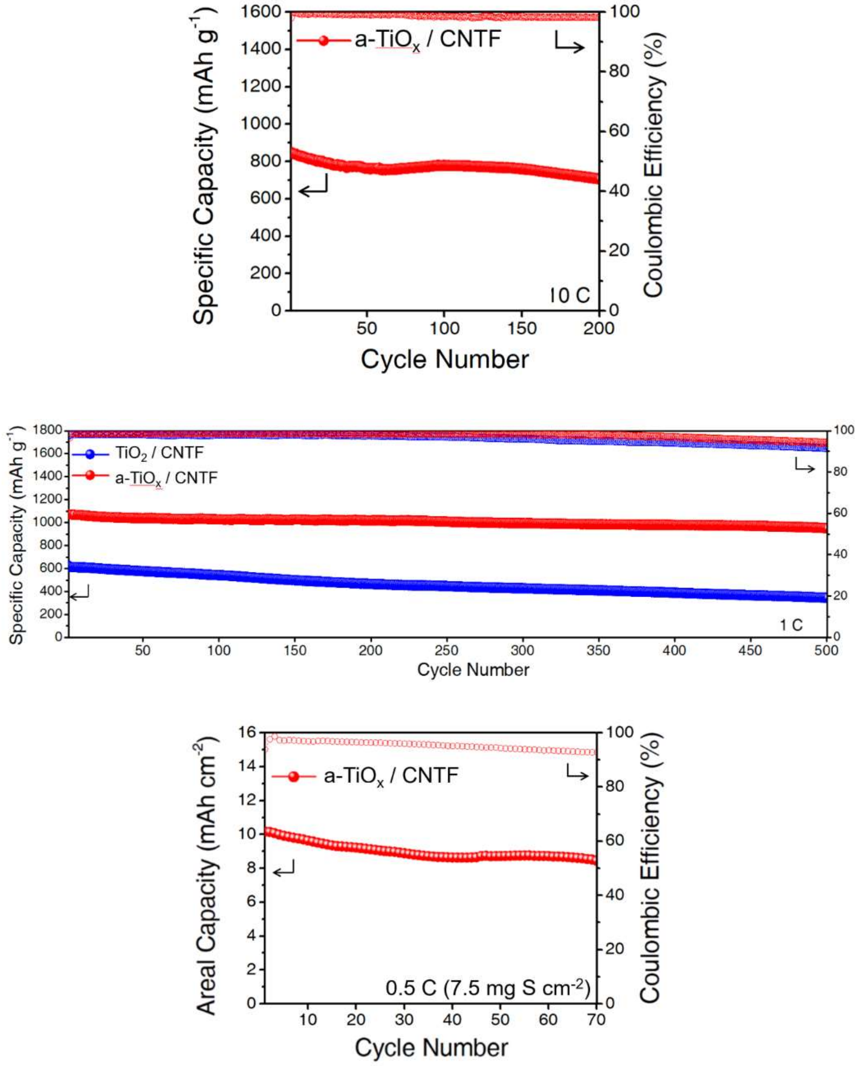


**Figure S9.** Coulombic efficiency according to charge/discharge cycle for (top) a-$TiO_x$/CNTF cell (10 C), (middle) $TiO_2$ / CNTF and a-$TiO_x$ / CNTF cathode cells (1 C), (bottom) a-$TiO_x$ / CNTF cathode cells (0.5 C, 7.5 mg S $cm^{-2}$)

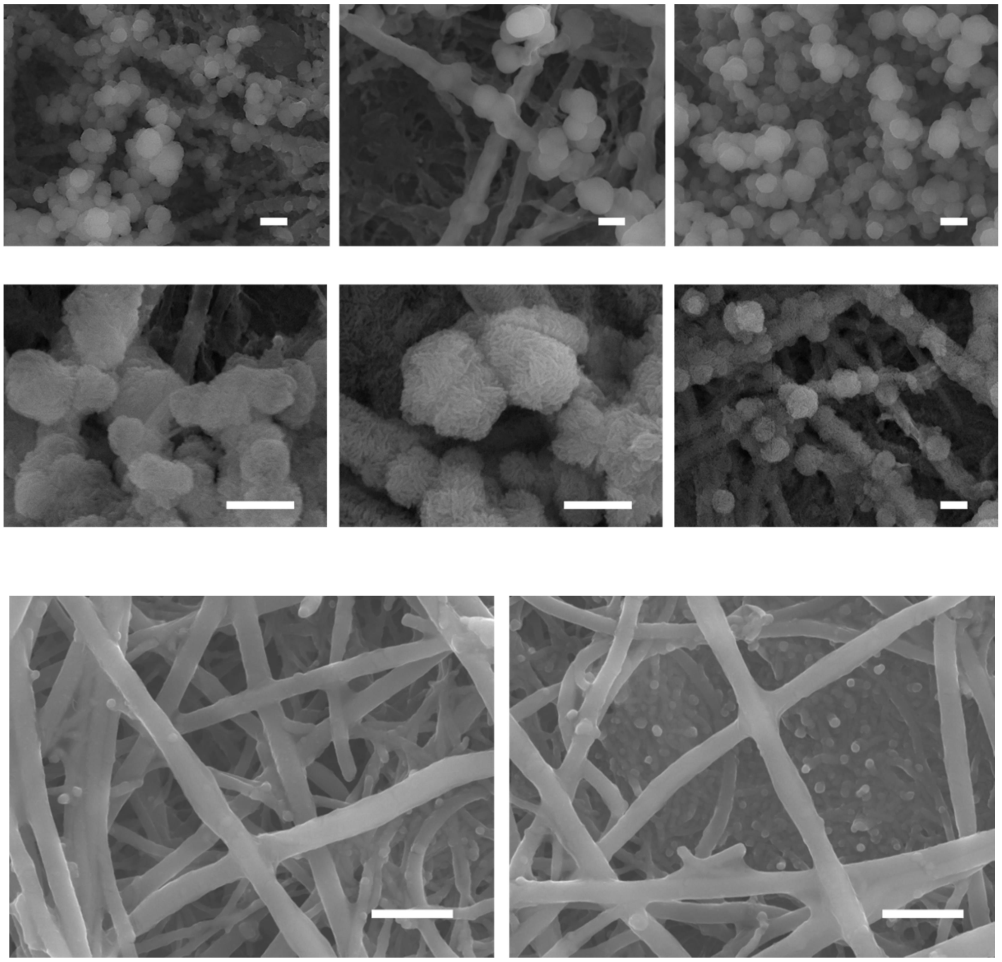

**Figure S10.** SEM images of the cathode after full discharge in the a-TiOx/CNTF cathode cell under constant current conditions of (Top panel) 0.2 C and (Middle panel) 10 C, respectively. Note the growth of particulate $Li_2S$ even at high rates of 10 C. The particulate $Li_2S$, similar to the $Li_2S$ form obtained in the constant voltage discharge experiment, is observed. (scale bar: 500 nm) (Bottom panel) SEM images of the cathode after full discharge in the $TiO_2$/CNTF cathode cell under constant current conditions of (Left) 0.2 C and (Right) 10 C, respectively. (scale bar: 1 μm)

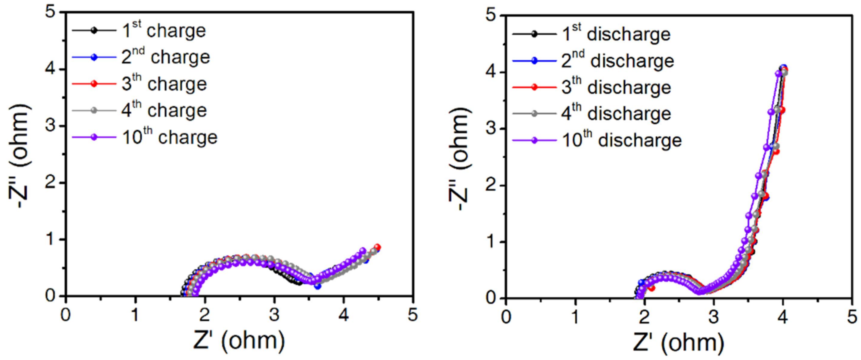


**Figure S11.** Operando electrochemical impedance spectroscopy (EIS) results for Li-S cells with high sulfur loading, multilayer a-$TiO_x$/CNTF cathode. Nyquist plots for discharge and charge cycles are displayed on the Left and Right, respectively. The semicircle is due to the charge transfer resistance at the electrode/electrolyte interface. During the discharge and charge cycles, this resistance value remains constant. This indicates that reversible sulfur conversion occurs in the multilayer a-$TiO_x$/CNTF cathode.

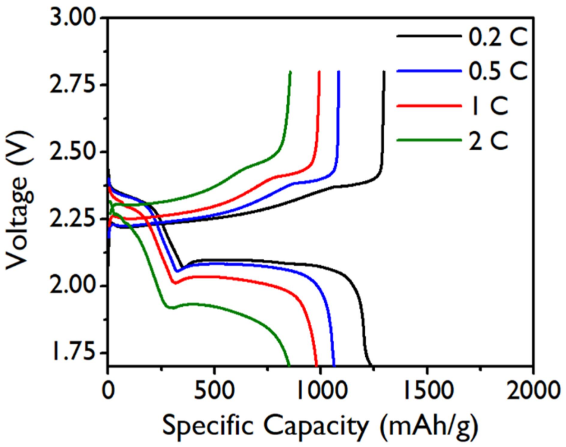


**Figure S12**. Voltage profile of a-$TiO_x$/CNTF electrode with high sulfur loading of 5 mg S/$cm^2$. At 0.2 C, the polarization shows a low value of 150 mV, indicating a reversible charge/discharge. At 1 C and 2 C, the polarization values are 250 mV and 400 mV, respectively. Stable polarization is exhibited even at the C-rate, which is increased by 5 and 10 times from 0.2 C.

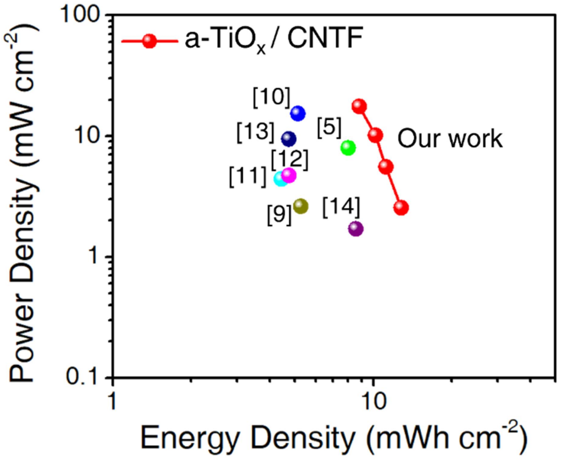


**Figure S13.** Ragone plots of cell performance obtained from previous metal compound/carbon composite electrodes and a-$TiO_x$/CNTF electrodes.[5, 9-14] Our results were calculated from the capacity obtained at various C-rates between 0.2 C – 2 C in a high-loading cathode of 5 mg S $cm^{-2}$.

**Table S1.** Sulfur utilization achieved in lithium-sulfur batteries using various metal compound cathodes.

| Cathode material | C-rate | Sulfur utilization (%) | Specific capacity (m Ah $g^{-1}$) | Sulfur ratio in cathode (%) |
|---|---|---|---|---|
| Our work | 0.2 C | 90 % | 1501 | 45 |
| LiBr salt anion electrolyte/carbon paper[15] | 0.2 C | 87 % | 1449 | 20 |
| Flower-like S/$Li_2S$ carbon fiber[16] | 0.2 C | 90 % | 1500 | 64 |
| 1,3-dimethyl-2-imidazolidinone electrolyte/carbon nanofiber[17] | 0.1 C | 73 % | 1225 | 49 |
| $CeO_2$/porous N-rich carbon nanosphere[18] | 0.2 C | 82 % | 1368 | 56 |
| Yolk-Shelled C@$Fe_3O_4$ nanobox[19] | 0.1 C | 82 % | 1366 | 64 |
| Hollow multi-shelled $TiO_{2-x}$[20] | 0.2 C | 76 % | 1277 | 42 |
| CNT/$TiO_{2-x}$[21] | 0.2 C | 69 % | 1149 | 56 |

**Table S2.** Areal capacities at high C-rates in recent results of introducing metal compound/carbon host for lithium-sulfur batteries.

| Cathode material | C-rate | Areal capacity (m Ah $cm^{-2}$) | Sulfur ratio in whole cathode (%) |
|---|---|---|---|
| Our work | 2 C | 4.3 | 45 |
| Walnut-shaped $VS_4$ nanosites combine with carbon nanotubes[5] | 1 C | 3.9 | N/A |
| Cobalt Clusters in Nitrogen-Doped Porous Carbon[9] | 0.5 C | 2.565 | 60 |
| Fe-N-Carbon microsphere[10] | 3 C | 2.5 | 48 |
| Paramontroseite $VO_2$ / N-Carbon nanotube[11] | 1 C | 2.16 | N/A |
| $Co_9S_8$/CoO heterostructure[12] | 1 C | 2.31 | 62 |
| $Fe_2N$@C Yolk-Shell nanobox[22] | 0.5 C | 4.2 | 64 |
| $WS_2$ nanosheets/carbon cloth[23] | 0.5 C | 1.42 | 11 |
| $FeCo_2S_4$/Carbon cloth[24] | 2 C | 2.17 | 17.6 |
| $TiNb_2O_7$/Carbon cloth[25] | 1 C | 1.8 | 44 |

References

[1] S. Amano, D. Bogdanovski, H. Yamane, M. Terauchi, R. Dronskowski, *Angew. Chem.* **2016**, *128*, 1684.

[2] S. Huang, Y. Von Lim, X. Zhang, Y. Wang, Y. Zheng, D. Kong, M. Ding, S. A. Yang, H. Y. Yang, *Nano Energy* **2018**, *51*, 340.

[3] L. Kong, X. Chen, B. Q. Li, H. J. Peng, J. Q. Huang, J. Xie, Q. Zhang, *Adv. Mater.* **2018**, *30*, 1705219.

[4] W. Yang, J. Xiao, Y. Ma, S. Cui, P. Zhang, P. Zhai, L. Meng, X. Wang, Y. Wei, Z. Du, *Adv. Energy Mater.* **2019**, *9*, 1803137.

[5] S. Wang, H. Chen, J. Liao, Q. Sun, F. Zhao, J. Luo, X. Lin, X. Niu, M. Wu, R. Li, *ACS Energy Lett.* **2019**, *4*, 755.

[6] H. Zhang, M. Zou, W. Zhao, Y. Wang, Y. Chen, Y. Wu, L. Dai, A. Cao, *ACS Nano* **2019**, *13*, 3982.

[7] Z.-L. Xu, S. Lin, N. Onofrio, L. Zhou, F. Shi, W. Lu, K. Kang, Q. Zhang, S. P. Lau, *Nat. Commun.* **2018**, *9*, 1.

[8] Z. Ye, Y. Jiang, L. Li, F. Wu, R. Chen, *Adv. Mater.* **2020**, *32*, 2002168.

[9] R. Wang, J. Yang, X. Chen, Y. Zhao, W. Zhao, G. Qian, S. Li, Y. Xiao, H. Chen, Y. Ye, *Adv. Energy Mater.* **2020**, *10*, 1903550.

[10] W.-G. Lim, Y. Mun, A. Cho, C. Jo, S. Lee, J. W. Han, J. Lee, *ACS Nano* **2018**, *12*, 6013.

[11] S. Wang, J. Liao, X. Yang, J. Liang, Q. Sun, J. Liang, F. Zhao, A. Koo, F. Kong, Y. Yao, *Nano Energy* **2019**, *57*, 230.

[12] N. Wang, B. Chen, K. Qin, E. Liu, C. Shi, C. He, N. Zhao, *Nano Energy* **2019**, *60*, 332.

[13] L. Peng, Z. Wei, C. Wan, J. Li, Z. Chen, D. Zhu, D. Baumann, H. Liu, C. S. Allen, X. Xu, *Nat. Catal.* **2020**, *3*, 762.

[14] J. Cai, J. Jin, Z. Fan, C. Li, Z. Shi, J. Sun, Z. Liu, *Adv. Mater.* **2020**, *32*, 2005967.

[15] H. Chu, H. Noh, Y.-J. Kim, S. Yuk, J.-H. Lee, J. Lee, H. Kwack, Y. Kim, D.-K. Yang, H.-T. Kim, *Nat. Commun.* **2019**, *10*, 1.

[16] H. Pan, J. Chen, R. Cao, V. Murugesan, N. N. Rajput, K. S. Han, K. Persson, L. Estevez, M. H. Engelhard, J.-G. Zhang, *Nat. Energy* **2017**, *2*, 813.

[17] M. Baek, H. Shin, K. Char, J. W. Choi, *Adv. Mater.* **2020**, *32*, 2005022.

[18] L. Ma, R. Chen, G. Zhu, Y. Hu, Y. Wang, T. Chen, J. Liu, Z. Jin, *ACS Nano* **2017**, *11*, 7274.

[19] J. He, L. Luo, Y. Chen, A. Manthiram, *Adv. Mater.* **2017**, *29*, 1702707.

[20] E. H. M. Salhabi, J. Zhao, J. Wang, M. Yang, B. Wang, D. Wang, *Angew. Chem.* **2019**, *131*, 9176.

[21] Y. Wang, R. Zhang, J. Chen, H. Wu, S. Lu, K. Wang, H. Li, C. J. Harris, K. Xi, R. V. Kumar, *Adv. Energy Mater.* **2019**, *9*, 1900953.

[22] W. Sun, C. Liu, Y. Li, S. Luo, S. Liu, X. Hong, K. Xie, Y. Liu, X. Tan, C. Zheng, *ACS Nano* **2019**, *13*, 12137.

[23] T. Lei, W. Chen, J. Huang, C. Yan, H. Sun, C. Wang, W. Zhang, Y. Li, J. Xiong, *Adv. Energy Mater.* **2017**, *7*, 1601843.

[24] B. Guo, S. Bandaru, C. Dai, H. Chen, Y. Zhang, Q. Xu, S. Bao, M. Chen, M. Xu, *ACS Appl. Mater. Interfaces* **2018**, *10*, 43707.

[25] X. Zhou, P. Zeng, H. Yu, C. Guo, C. Miao, X. Guo, M. Chen, X. Wang, *ACS Appl. Mater. Interfaces* **2021**.
